# Light-Driven Self-Pulsating Hydrogel with a Sliding-Delay Mechanism for Micro-Actuation and Microfluidic Applications

**DOI:** 10.3390/mi17040503

**Published:** 2026-04-21

**Authors:** Xingui Zhou, Huailei Peng, Yunlong Qiu, Cong Li

**Affiliations:** School of Civil Engineering, Anhui Jianzhu University, Hefei 230601, China; zhouxingui@ahjzu.edu.cn (X.Z.); phl@stu.ahjzu.edu.cn (H.P.); ylqiu@stu.ahjzu.edu.cn (Y.Q.)

**Keywords:** light-responsive hydrogel, self-pulsating, photoisomerization, time delay

## Abstract

Light-responsive hydrogel-based oscillators typically exhibit small oscillation amplitudes because solvent diffusion is intrinsically slow, and their dependence on external periodic light modulation further results in limited amplitude, poor stability, and insufficient autonomy. Inspired by the trigger and sliding mechanism of the ancient crossbow, this study introduces an innovative system that integrates a sliding-block mechanism with time-delay feedback, breaking from conventional approaches that rely on hydrogel inertia or external modulation, within a purely theoretical and simulation-based framework. By establishing a nonlinear dynamic model coupling solvent diffusion, photoisomerization, and optical attenuation, this research shows through numerical simulations that the system can exhibit two distinct modes under constant illumination: a stable state and a self-sustained oscillatory state. The model predicts that the oscillation frequency can be flexibly tuned by varying key parameters, including the crosslinking density, Flory–Huggins interaction parameters of the spiropyran and hydrophilic polymer, ring-opening reaction rate, light intensity, fraction of light-sensitive molecules, and sliding displacement, whereas the initial absorption coefficient has only a minor influence. The slider displacement is also identified as an effective means to regulate the oscillation amplitude. Furthermore, the expansion force at the container bottom is predicted to oscillate synchronously with the hydrogel’s volume change. This theoretical framework represents a paradigm shift from “static small deformation” to “dynamic large-amplitude oscillation”, significantly enhancing the mechanical responsiveness of the material. This work provides a novel and controllable strategy for the conceptual design of autonomous light-driven micromechanical systems.

## 1. Introduction

Self-excited motion is a widely occurring and functionally significant dynamic process, observed in engineering systems such as the flutter of wind turbine blades and sustained oscillation of mechanical pendulums [1,2], as well as in natural phenomena such as the regular beating of the human heart and the periodic flapping of insect wings [3,4]. Its distinguishing feature is that it can operate autonomously for extended periods without requiring external periodic excitation or a fixed power source [5]. This motion is sustained by nonlinear internal coupling mechanisms that enable interactions with the surroundings, continuously harvesting and converting ambient energy to offset unavoidable dissipation [6,7,8], thereby maintaining stable and predictable periodic oscillations. Intrinsic system conditions primarily set the frequency and amplitude of the self-excited motion [9,10], offering excellent controllability, repeatability, and tunability. Compared with conventional forced oscillation systems [11,12], self-oscillation significantly reduces dependence on external driving devices, helping to simplify system structures and lower energy consumption. Moreover, such systems exhibit greater autonomy and enhanced operational stability compared to conventional forced oscillators. Benefiting from autonomous operation, reduced energy demand, and enhanced stability, self-oscillating systems possess considerable promise for practical implementation, including warning indicators [13], actuator systems [14], soft robotics [15], and biomedical engineering [16]. Such systems enable the conversion of environmental energy, autonomous actuation, periodic motion, and rhythmic output. When combined with smart materials such as light-responsive hydrogels [17], self-oscillation is poised to open up new avenues in biomimetics [18], microfluidics [19], and actuator systems [20].

Recently, self-sustained oscillatory systems utilizing active materials have garnered considerable attention due to their ability to continuously generate mechanical output and deformation without external periodic driving. Through internal nonlinear coupling mechanisms [21,22], these materials can convert continuous environmental energy into stable periodic mechanical responses, thereby achieving self-excited oscillation. The system exploits the self-regulating characteristics of its materials and structures to autonomously adjust oscillatory behavior when exposed to external cues, including light, temperature, moisture, or chemical gradients [23,24,25]. When the material responds to external stimuli, it undergoes swelling, contraction, or deformation, and through mechanical, chemical, and thermal feedback mechanisms, converts the input energy efficiently into repeatable and controllable output behaviors, thereby achieving efficient energy utilization and adaptive dynamic regulation. Various strategies have been devised to induce self-oscillatory motion across different materials [26,27,28,29], including rolling [30,31,32], swinging [33,34,35,36,37], rotating [38,39], vibrating [40,41], buckling [42,43,44], jumping [45,46,47], swimming [48], sliding [49], striking [50], launching [51], pendulum motion [52], peeling [53], galloping [54], synchronization [55], and flipping [56]. These mechanisms have been extensively studied and applied across a wide range of systems.

In recent years, responsive hydrogels have emerged as smart materials that can undergo pronounced physical and chemical changes in response to external cues [57,58,59,60], including light [61,62,63,64], temperature [65,66,67,68], pH [69], humidity [70,71], or chemical gradients [72]. Their main characteristics include reversible volume changes, tunable mechanical properties, and morphological reconfiguration capabilities [73,74]. The internal network structure of hydrogels responds to external stimuli, inducing macroscopic dimensional changes via water absorption and swelling or water expulsion and contraction [75,76], along with changes in mechanical characteristics, including strength and elastic modulus [77,78]. A wide range of hydrogel materials, including agarose and alginate, have been extensively employed in microfluidic platforms due to their biocompatibility and tunable transport properties [79,80,81]. Light-responsive hydrogels have garnered significant interest owing to their non-contact controllability and high spatial tunability. Under light stimulation, these materials can undergo reversible volume changes, modulate mechanical properties, and switch between hydrophilic and hydrophobic states [82], thereby efficiently converting external light energy into controllable mechanical or chemical responses. These characteristics suggest that light-responsive hydrogels may have potential applicability in biomedical engineering, smart actuation, and microfluidic systems. Recent studies have primarily focused on improving the light-sensitive components to enhance response efficiency, increase the reversibility and speed of volume and mechanical property changes [83], and achieve precise control over material behavior by finely tuning the intensity and wavelength of light [84].

**Figure 1:**
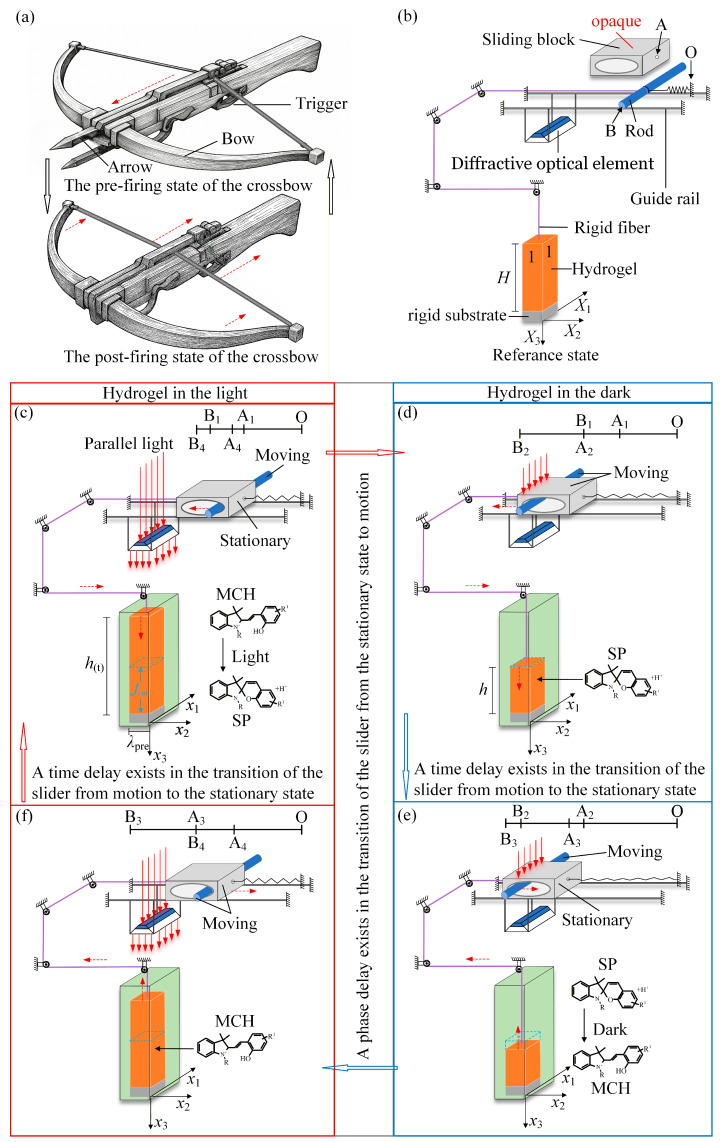
Dynamic model of the self-pulsating light-responsive hydrogel under continuous illumination. (**a**) The ancient crossbow employs a clearance structure between the arrow, bow, and trigger to achieve delayed release and instantaneous energy output. (**b**) Reference state. (**c**) Initial state. (**d**) Dark state. (**e**) Phase-delayed contraction state. (**f**) Light state. During sustained light exposure, the light-responsive hydrogel contracts along the $x_{3}$-axis and dynamically adjusts the position of the sliding block, thereby forming a feedback loop with the light intensity that drives and sustains periodic oscillations in the system.

Light-responsive hydrogels have been extensively explored for constructing self-oscillating systems due to their ability to undergo swelling-deswelling transitions under light stimulation [85,86,87,88]. Traditional light-responsive hydrogels suffer from slow response constrained by molecular isomerization and solvent diffusion, strong dependence on the intensity and wavelength of external light, and difficulties in precisely controlling oscillation amplitude and frequency [89,90,91]. Furthermore, existing hydrogel-based self-oscillatory systems mainly rely on photothermal effects, chemical oscillators (e.g., BZ reactions), or feedback-controlled swelling mechanisms [92,93,94]. Photothermal systems are driven by thermal effects, and chemical oscillators depend on specific reaction conditions, while feedback-controlled systems often require external modulation or finely tuned parameters. Therefore, they still exhibit limitations in terms of autonomy and controllability. As shown in Figure 1a, the ancient crossbow used a clearance structure between the arrow, bow, and trigger to keep the arrow stationary during energy storage and prevent premature force transfer. Only when the trigger was released did the stored energy instantly drive the arrow. This free-travel design enabled controlled delay, maximal energy storage, and precise firing. Inspired by the ancient crossbow, this study proposes a geometrically designed light-responsive hydrogel self-pulsating system. Under constant illumination, we introduce a clearance slider mechanism and time-delay feedback: the slider provides mechanical constraint and feedback regulation, converting the swelling-deswelling of hydrogels into more stable and amplified periodic oscillations, while the time delay breaks the limitation of purely inertial dynamics and offers a new pathway for dynamic regulation. This combination effectively enhances mechanical output efficiency and enables control over oscillation direction and rhythm, allowing the system to maintain self-oscillation under steady illumination and significantly reducing reliance on external periodic signals or inertial effects. The proposed geometric control and delayed feedback strategy not only improve system robustness and reproducibility but also provides some reference for the design of self-driven systems [95] and intelligent devices [96,97]. Compared with existing hydrogel-based self-oscillatory systems, the geometrically regulated mechanism proposed in this work, based on a sliding-block constraint and time-delay feedback, enables self-sustained large-amplitude oscillations under constant illumination without relying on intrinsic chemical oscillations or external periodic inputs, thereby providing a new pathway for dynamic regulation in hydrogel systems.

This paper is structured as follows. Section 2 derives the governing equations of the hydrogel through a nonlinear dynamic model and introduces the corresponding solution methods. Section 3 presents the two dynamic states of the light-responsive hydrogel system and examines the mechanisms underlying the self-pulsating state. Section 4 analyzes the role of critical parameters in governing the frequency and amplitude of self-pulsating, and Section 5 analyzes the hydrogel-generated stress and swelling force during the contraction-expansion process.

## 2. Model and Theoretical Description

Here, we first formulate a model for a light-responsive hydrogel under continuous light irradiation. Based on a dynamic framework, we derive the governing equations for solvent diffusion, describe the interconversion between the two photoisomeric states of the photosensitive molecules, and present the nondimensional formulation and corresponding solution methods.

### 2.1. Dynamic Model of the Light-Responsive Hydrogel Under Continuous Illumination

Figure 1 illustrates the light-responsive hydrogel slider system inspired by the clearance structure of the ancient crossbow. The system consists of a light-responsive hydrogel fixed at the bottom onto a transparent rigid substrate and aligned parallel to the $x_{3}$-axis. Above the hydrogel is a diffractive optical element component suspended on a guide rail, which converts the line-shaped laser into an area beam. At the upper right of the light-responsive hydrogel is a slider that can move horizontally along the guide rail. Let the reference position of the slider on the guide rail be denoted as point A, and denote by Δ the displacement of the slider’s current position relative to point A, with $\bar{\Delta }=\Delta /H$, and its top surface is opaque. Inside the slider is a cylindrical rod that drives its movement, which can only slide horizontally. The rod’s position is denoted as point B. The hydrogel’s top is linked to a rigid fiber, one end of which is attached to the cylindrical rod, while the opposite side of the rod is connected to a spring whose other end is fixed to a support at point O. The system assumes continuity boundary conditions at both sides of the hydrogel to ensure that deformation occurs only along the $x_{3}$-axis. As shown in Figure 1b, let $X_{1}$ and $X_{2}$ represent the material coordinates within the plane of the dry gel layer, while $X_{3}$ denotes the material coordinate along the normal direction. In the reference state, the gel layer’s dry-state thickness is defined as H. In the absence of illumination, the isomeric forms within the hydrogel remain unchanged, and therefore the hydrogel does not undergo any deformation.

Figure 1c illustrates the hydrogel, together with its substrate, fixed inside a water-filled glass container. The dry gel layer is initially subjected to a transverse pre-stretch, characterized by a prescribed stretch ratio $\lambda_{\mathrm{pre}}$. The hydrogel is then allowed to fully absorb water until it reaches saturation, at which point its height h(t) is defined as the initial height $h_{0}$. Continuous line-shaped illumination is applied to the upper part of the hydrogel, with the light directed parallel to both the $x_{3}$-axis and the gel layer. A diffractive optical element (DOE) mounted on the guide rail transforms the line beam into a planar beam, ensuring uniform illumination over the top surface of the light-responsive hydrogel. As light penetrates the gel layer, a photochemical reaction first occurs at the end nearest the light. The internal isomers undergo a structural transition from hydrophilic to hydrophobic. With increasing hydrophobicity, the hydrogel expels water, leading to a marked volume reduction. Meanwhile, as light propagates through the hydrogel, photosensitive molecules absorb the light, accompanied by a decrease in the absorption coefficient, thereby altering the internal light distribution. As the volume shrinks, the rod is pulled toward the left end of the slider by the fiber. At this stage, the rod is at point B1, the slider at point A1, and the spring is stretched.

Figure 1d illustrates that as the volume continues to decrease, the cylindrical rod begins to drive the sliding block, and the slider exhibits a phase delay when transitioning from rest to motion. At this point, the rod is located at point B2, and the slider is positioned at point A2. The height of the gel layer during deformation is denoted as h, and it is defined that $\lambda_{H}=h/H$. When the sliding block moves directly beneath the continuous illumination and blocks the light, the hydrogel loses exposure to light. Driven by the internal chemical potential, the light-responsive hydrogel continues to contract until all internal isomer molecules are fully converted into the SP form. Once the isomers switch from hydrophobic to hydrophilic, the hydrogel absorbs water and swells, thereby driving the rod to move to the right, as depicted in Figure 1e. At this stage, the rod is at point B3, and the corresponding position of the slider is point A3. Figure 1f illustrates that as the hydrogel continues to swell, driven by the spring, the rod first displaces to the right end of the slider and then propels the slider to move synchronously. At this moment, the rod is at point B4, and the slider is at point A4. This process continues until the mechanism returns to the configuration illustrated in Figure 1c, thereby completing one full cycle of self-pulsating of the light-responsive hydrogel-slider system. Meanwhile, the threshold difference introduced by the slider delays the switching of illumination, so that the system continues to be driven even during the phase when it should be suppressed, which in turn amplifies the oscillation amplitude. This switching condition is now explicitly formulated as: illumination is ON when $J>J_{\mathrm{crit}}$ and OFF when $J\leq J_{\mathrm{crit}}$. The critical volume $J_{\mathrm{crit}}$ is determined by the slider displacement Δ and the clearance geometry. For the parameter values used in this study, $J_{\mathrm{crit}}$ = 2.35.

### 2.2. Photochemical Dynamics

Under uniform continuous illumination, the light intensity within the light-responsive hydrogel is non-uniformly distributed. This process is described by the Beer-Lambert law [98], and taking into account the light attenuation that occurs as the hydrogel absorbs light, we obtain:

(1) $$\frac{\partial I\left(X_{3},t\right)}{\partial X_{3}}=-A\left(X_{3},t\right)\lambda_{3}\left(X_{3},t\right)I\left(X_{3},t\right).$$

In Equation (1), $A(X_{3},t)$ is the absorption coefficient at material position $X_{3}$ and time t, and $I(X_{3},t)$ represents the light intensity. It is assumed:

(2) $$A\left(X_{3},t\right)=A_{0}\left(1-r_{\mathrm{SP}}\right),$$

here, $A_{0}$ is the initial absorption coefficient, $A_{0}$ corresponding to the proportion of light-sensitive SP in the hydrogel, $r_{\mathrm{SP}}\left(X_{3},t\right)$ is the fraction of light-responsive molecules (SP) in the hydrogel. And the vertical stretch $\lambda_{3}(X_{3},t)$ is

(3) $$\lambda_{3}\left(X_{3},t\right)=\frac{\partial x_{3}\left(X_{3},t\right)}{\partial X_{3}}.$$

Therefore, Equation (1) can be derived into the distribution equation of the light intensity I:

(4) $$\frac{\partial I}{\partial X_{3}}=-A_{0}\lambda_{3}\left(1-r_{\mathrm{SP}}\right)I.$$

$r_{\mathrm{MCH}}(X_{3},t)$ denotes the fraction of light-sensitive MCH in the hydrogel, and $k_{c}$ is the ring-closing reaction rate, which is governed by the quantum yield of SP and the number of photons absorbed by MCH [99]. $k_{0}$ is the ring-opening reaction rate, determined by pH and temperature [100]. To describe the interconversion between the MCH and SP isomeric forms, we adopt the differential equation for the photochemical reaction of $r_{\mathrm{SP}}$ [91]:

(5) $$\frac{\partial r_{\mathrm{SP}}}{\partial t}=-k_{c}Ir_{\mathrm{MCH}}-k_{0}r_{\mathrm{SP}},$$

since here $r_{\mathrm{SP}}+r_{\mathrm{MCH}}=1$, Equation (5) can be expressed as:

(6) $$\frac{\partial r_{\mathrm{SP}}}{\partial t}=k_{c}I\left(1-r_{\mathrm{SP}}\right)-k_{0}r_{\mathrm{SP}}.$$

Since the hydrogel itself is nearly incompressible, its macroscopic volume change is primarily attributed to variations in internal solvent content. Therefore, under uniform continuous illumination, we denote the hydrogel height as h and its width as a constant $\lambda_{\mathrm{pre}}$. For regions within the hydrogel where isomeric conversion occurs, the volume change in the hydrogel can be expressed as:

(7) $$J=\det\;F=1+\Omega C\left(x,t\right)={\lambda_{\mathrm{pre}}}^{2}\lambda_{3},$$

where J is the volumetric deformation of the hydrogel, F denotes the deformation gradient tensor of the hydrogel, C represents the solvent concentration, and Ω is the volume of each solvent molecule.

### 2.3. Diffusion Dynamics

In studying deformation of hydrogels, the composition of the free energy is crucial for understanding their deformation response and swelling behavior. The free energy of a light-responsive hydrogel is composed of the elastic energy $\psi_{\mathrm{el}}$ and the mixing free energy $\psi_{\mathrm{mix}}$. During both thermodynamic equilibrium and non-equilibrium processes, the minimization of free energy drives the hydrogel’s volume change and structural evolution. Accordingly, we can introduce the free energy function ψ [101,102]:

(8) $$\psi =\psi_{\mathrm{elastic}}+\psi_{\mathrm{mix}C}.$$

In Equation (8), $\psi_{\mathrm{elastic}}$ and $\psi_{\mathrm{mix}C}$ can be expressed as:

(9) $$\psi_{\mathrm{elastic}}=\frac{1}{2}NkT(I_{1}-3-2\log\;J),$$

(10) $$\psi_{\mathrm{mixC}}=\frac{kT}{v}\left[\left(1-\phi )In(1-\phi )+\chi \phi (1-\phi \right)\right],$$

where N denotes the crosslinking density, kT represents the temperature, NkT is the shear modulus, $I_{1}=\mathrm{tr}\left(FF^{T}\right)$, v is the volume of a segment, ϕ is the polymer volume fraction, and χ is Flory–Huggins interaction parameter of hydrophilic polymers. By combining Equations (9) and (10), Equation (8) can be written as:

(11) $$\begin{array}{l} \psi =\frac{1}{2}NkT\left(I1-3-2\log\;J\right)\\ +\frac{KT}{\Omega }\left\{\Omega \left[\log\left(\frac{\Omega C}{1+\Omega C}\right)\right]+\frac{\chi \left(1-f\right)\left(\Omega C+f\right)+\chi_{\mathrm{SP}}f\left(1+\Omega C-f\right)}{1+\Omega C},\right\} \end{array}$$

here, $\chi_{\mathrm{SP}}$ denotes the Flory–Huggins interaction parameter of the hydrophilic SP, and f represents the ratio of light-sensitive molecules to the polymer.

To further characterize solvent diffusion in hydrogels, we propose the concept of chemical potential, which quantifies the thermodynamic potential-energy gradient of solvent molecules and acts as the primary driving force for swelling and solvent migration. Under continuous illumination, the chemical potential within the hydrogel increases, promoting solvent expulsion; conversely, when the light intensity decreases, solvent re-enters the network. In this study, we assume that solvent molecules inside the hydrogel remain in local chemical equilibrium with those entering or leaving the system. Moreover, the chemical potential is directly obtained from the hydrogel’s free energy expression. Based on these considerations, the solvent transport equation is given by:

(12) $$\mu =\frac{\partial \psi }{\partial C}+p\Omega ,$$

with p representing Lagrange multiplier.

By combining with Equation (11), we obtain:

(13) $$\mu =kT\left[\log\left(\frac{\Omega C}{1+\Omega C}\right)+\frac{1}{1+\Omega C}+\frac{\chi {\left(1+f\right)}^{2}+\chi_{\mathrm{sp}}f^{2}}{{\left(1+\Omega C\right)}^{2}}\right]+p\Omega ,$$

here, we assume that:

(14) $$f=f_{\max}r_{\mathrm{sp}}\left(x\right).$$

Equation (13) can be written as:

(15) $$\begin{array}{l} \mu =kT\left[\log\left(\frac{\Omega C}{1+\Omega C}\right)+\frac{1}{1+\Omega C}+\frac{\chi {\left(1+f_{\max}r_{\mathrm{sp}}\right)}^{2}+\chi_{\mathrm{sp}}f_{\max}{r_{\mathrm{sp}}}^{2}}{{\left(1+\Omega C\right)}^{2}}\right]\\ +p\Omega . \end{array}$$

The deformation behavior of the light-responsive hydrogel is primarily driven by the spatiotemporal variations in solvent concentration. Changes in solvent concentration directly alter the swelling equilibrium between polymer chains within the gel network, leading to local expansion or contraction. When illumination induces isomerization reactions, the structure of the light-sensitive molecules inside the gel changes, thereby modifying the local hydrophilicity and generating spatial gradients in solvent concentration. These concentration gradients not only drive solvent migration within the gel but also induce non-uniform swelling, resulting in a complex mechanical response. Accordingly, the mass balance equation for the solvent is given by:

(16) $$\frac{\partial C}{\partial t}=-\nabla \cdot h,$$

in which, h is the flux of solvent diffusion, and the h is related to the gradient of μ [103]:

(17) h=−M·∇μ,

here, μ is the chemical potential, M can be expressed as:

(18) $$M=\frac{D}{\Omega kT}\left(J-1\right)F^{-1}F^{-T}.$$

By combining Equations (17) and (18), Equation (16) can be derived as:

(19) $$\frac{\partial C}{\partial t}=\nabla \cdot \left[\frac{D}{\Omega kT}\left(J-1\right)F^{-1}F^{-T}\cdot \nabla \mu \right],$$

since the chemical potential *μ* has already been established in Equation (15), Equation (19) can now be analyzed and solved.

Given that localized reorganization of solvent species occurs far more rapidly than long-range diffusion, the mechanical behavior of the hydrogel is expressed using the first Piola-Kirchhoff stress tensor [91]:

(20) $$P=\frac{\partial \psi }{\partial F}-pJF^{-T}.$$

Based on Equation (20), the Cauchy stress can be calculated as:

(21) $$\sigma =\frac{PF^{T}}{J}=\frac{1}{J}F^{T}\cdot \frac{\partial \psi }{\partial F}-pI.$$

In the equation, I is the identity matrix, and P represents the net water pressure. By combining with Equations (7) and (8), Equation (21) can be rewritten as:

(22) $$\sigma =\frac{1}{J}NkTFF^{T}-pI.$$

Therefore, the lateral stress component can be expressed as:

(23) $$\sigma_{1}=\sigma_{2}=\frac{NkT}{J}({\lambda_{3}}^{2}-1)-P.$$

Since the hydrogel layer is considered to be in mechanical equilibrium during solvent migration, $\sigma_{3}=0$, and the hydrostatic pressure can be derived as:

(24) $$p=NkT\left(1+\Omega C-\frac{1}{1+\Omega C}\right).$$

Using Equation (24), Equation (15) can be rewritten as:

(25) $$\begin{array}{ll} \mu \left(x,t\right) & =kT\left\{\log\left[\frac{\Omega C\left(x,t\right)}{1+\Omega C\left(x,t\right)}\right]+\frac{1}{1+\Omega C\left(x,t\right)}+\frac{\chi {\left[1+f_{\max}r_{\mathrm{SP}}\left(x\right)\right]}^{2}+\chi_{\mathrm{SP}}{\left[f_{\max}r_{\mathrm{SP}}\left(x\right)\right]}^{2}}{{\left(1+\Omega C\left(x,t\right)\right)}^{2}}\right\}\\ & +NkT\left[1+\Omega C\left(x,t\right)-\frac{1}{1+\Omega C\left(x,t\right)}\right]\Omega . \end{array}$$

Equations (18) and (25) are the governing equations for the solvent diffusion process, and the diffusion process depends on the concentration C.

Based on this, the swelling force induced by the hydrogel’s expansion-contraction can be calculated from the lateral stress as:

(26) $$F(t)=w\int_{0}^{H}\sigma_{1}\left(X_{3},t\right)\lambda_{3}\left(X_{3},t\right)dX_{3},$$

in which w denotes the breadth of the substrate.

### 2.4. Dimensionless and Solution

For convenience in calculation, we introduce the dimensionless parameters $\bar{\mu }=\mu /kT$, $\bar{C}=\Omega C$, $\bar{N}=N\Omega$, $\bar{t}=Dt/H^{2}$, ${\bar{X}}_{3}=X_{3}/H$, ${\bar{k}}_{0}=k_{0}H^{2}/D$, $\bar{I}=k_{c}IH^{2}/D$, ${\bar{A}}_{0}=A_{0}H$, $\bar{\sigma }=\sigma \Omega /kT$, $\bar{p}=p\Omega /kT$, $\bar{F}=F\Omega /wHkT$, $\bar{\Delta }=\Delta /H$. Under continuous illumination, the nondimensional form of the light intensity distribution equation within the hydrogel (Equation (4)) is:

(27) $$\frac{\partial \bar{I}}{\partial \bar{x}}=-{\bar{A}}_{0}\left(1-r_{\mathrm{SP}}\right)\bar{I}.$$

Equation (6) describes the light-sensitive fraction of SP in the hydrogel, and its nondimensional form is:

(28) $$\frac{\partial r_{\mathrm{SP}}}{\partial \bar{t}}=\bar{I}\left(1-r_{\mathrm{SP}}\right)-{\bar{k}}_{0}r_{\mathrm{SP}}.$$

According to Equation (7), the correlation between the solvent content and vertical extension can be cast into a dimensionless form as presented below:

(29) $$1+\bar{C}=\lambda_{\mathrm{pre}}^{2}\lambda_{3}.$$

Equation (11), describing solvent diffusion in the hydrogel, is expressed in dimensionless form as follows:

(30) $$\frac{\partial \bar{C}}{\partial \bar{t}}=-\frac{\partial }{\partial {\bar{X}}_{3}}\left(\frac{\bar{C}\partial \bar{\mu }}{\lambda_{3}^{2}\partial {\bar{X}}_{3}}\right).$$

Equation (23), describing lateral stress, is expressed in dimensionless form as follows:

(31) $${\bar{\sigma }}_{1}={\bar{\sigma }}_{2}=\frac{1}{J}(\lambda_{3}^{2}-1)-\bar{p}.$$

The nondimensional form of the chemical potential equation within the hydrogel (Equation (25)) is:

(32) $$\bar{\mu }=\log\left(\frac{\bar{C}}{1+\bar{C}}\right)+\frac{1}{1+\bar{C}}+\frac{\chi {\left(1+f_{\max}r_{\mathrm{SP}}\right)}^{2}+\chi_{\mathrm{SP}}{\left(f_{\max}r_{\mathrm{SP}}\right)}^{2}}{{\left(1+\bar{C}\right)}^{2}}+\bar{N}\left(1+\bar{C}-\frac{1}{1+\bar{C}}\right).$$

The nondimensional form of the hydrogel swelling force calculation equation (Equation (26)) is:

(33) $$\bar{F}(\bar{t})=\int_{0}^{1}{\bar{\sigma }}_{1}({\bar{X}}_{3},\bar{t})\lambda_{3}({\bar{X}}_{3},\bar{t})d{\bar{X}}_{3}.$$

Prior to the initial state, we assume that the hydrogel is in the dark, with all isomers in the MCH form and no isomerization to the SP form yet initiated. Therefore, the preliminary conditions are defined as:

(34) $$I\left({\bar{X}}_{3},0\right)=0,$$

(35) $$r_{\mathrm{SP}}\left({\bar{X}}_{3},0\right)=0,$$

(36) $${\bar{r}}_{\mathrm{MCH}}\left({\bar{X}}_{3},0\right)=1,$$

(37) $$\bar{C}({\bar{X}}_{3},0)=C_{0},$$

(38) $$\lambda_{3}\left({\bar{X}}_{3},0\right)=\frac{h_{0}}{H}.$$

Since the top of the container is considered impermeable to water, the hydrogel’s top surface exhibits the following characteristics:

(39) $$\frac{\partial \bar{C}(0,\bar{t})}{\partial {\bar{X}}_{3}}=0,$$

(40) $$\frac{\partial \lambda_{3}(0,\bar{t})}{\partial {\bar{X}}_{3}}=0.$$

The computational steps are summarized below. When continuous light illuminates the hydrogel, the light intensity propagates within the gel and can be calculated using Equation (27). Under the influence of light, photochemical reactions occur, converting internal isomers from the MCH form to the SP form, with the SP distribution calculated via Equation (28). Subsequently, the chemical potential within the hydrogel is calculated using Equation (32), and the solvent concentration is obtained through Equation (30). Based on the computed solvent concentration, the volume change in the hydrogel is determined using Equation (29). Meanwhile, the movement of the slider adjusts the light illumination area based on the hydrogel’s volume changes, realizing a closed-loop feedback control between volume and light. Finally, the lateral stress is calculated via Equation (31) and substituted into Equation (33) to compute the swelling force resulting from the hydrogel’s expansion-contraction. It should be noted that the present model is established under several idealized assumptions. In particular, mechanical friction between the slider and the guide rail, fabrication-induced geometric imperfections, and optical misalignment are neglected. In addition, the hydrogel is assumed to be homogeneous, and long-term material degradation or fatigue effects are not considered. These simplifications enable a clearer identification of the intrinsic chemo–mechanical–optical coupling mechanism but may affect the quantitative accuracy of the predicted oscillatory behavior in practical implementations.

## 3. Two Motion States and Self-Pulsating Mechanism

Here, using the analysis for the solutions to the governing Equations (27)–(33), two motion states of the light-responsive hydrogel system are characterized: the stable and the self-pulsating state. The fundamental formation mechanisms corresponding to each motion state are also described in detail.

### 3.1. Stable State and Self-Pulsating State

To further investigate the self-pulsating state of the light-responsive hydrogel system, representative material properties and geometric parameters are provided in Table 1, along with the relevant dimensionless parameters shown in Table 2. In the subsequent sections, the specified parameter values will be employed to investigate the photochemical reaction behavior of the hydrogel subjected to continuous light irradiation. To facilitate assessment of the practical relevance of the predicted oscillations, we provide order-of-magnitude estimates relating the dimensionless parameters to physically measurable quantities. Based on the values in Table 1 and Table 2, the dry hydrogel thickness is H = 0.5 mm and the solvent diffusion coefficient is D = 3 × 10^−9^ m^2^/s. The characteristic diffusion time is therefore $\tau =H^{2}/D\approx 83$ s. A dimensionless time increment of $\Delta \bar{t}=1$ corresponds to approximately 83 s of physical time. The dimensionless oscillation frequencies reported in Section 4 (e.g., f≈0.15–0.25) therefore correspond to physical frequencies of approximately to 0.0018–0.0030 Hz, i.e., one oscillation cycle every 5–10 min. The critical volume threshold $J_{\mathrm{crit}}=2.35$ corresponds to a physical hydrogel thickness of approximately 1.2 mm when the in-plane pre-stretch $\lambda_{\mathrm{pre}}=1$. These estimates confirm that the predicted self-pulsating operates on time scales consistent with typical solvent diffusion in responsive hydrogels. The parameter values used in the simulations are selected from representative ranges reported in the literature for light-responsive hydrogels and are consistent with the thermodynamic framework described in Ref. [87].

**Figure 2:**
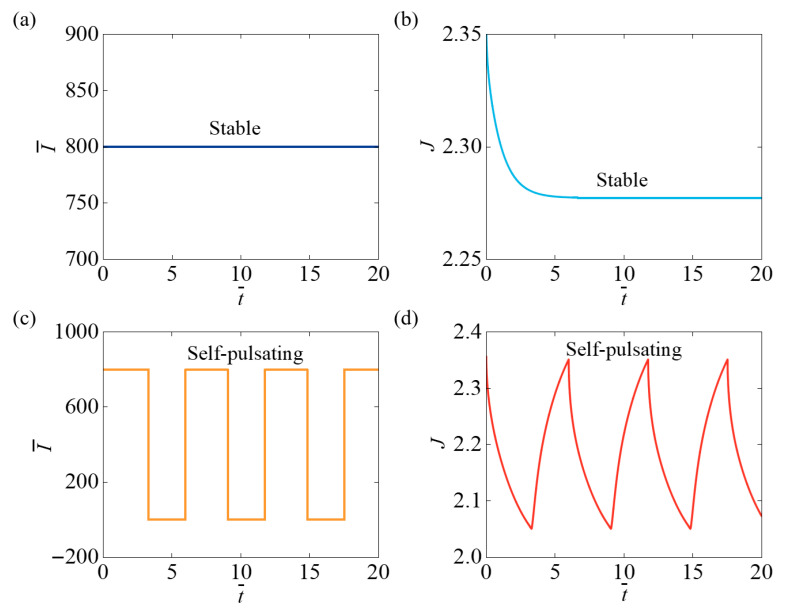
Two states of the light-responsive hydrogel. (**a**,**b**) show the time-course curves of light intensity and hydrogel volume ($\bar{N}=0.8$, χ=0.2). (**c**,**d**) depict the time-course curves of light intensity and hydrogel volume ($\bar{N}=0.2$, χ=0.4). Due to differences in material parameters, the system can enter two distinct motion states: the stable state and the self-pulsating state.

Figure 2 illustrates that, under specific parameter settings, the light-responsive hydrogel system shows two reaction states: the stable and the self-pulsating state. Figure 2a,b depict the stable state, where both the light intensity and hydrogel volume level off over time. The parameters are set as: $\bar{N}=0.8$, χ=0.2, $\chi_{\mathrm{SP}}=0.65$, ${\bar{A}}_{0}=1$, ${\bar{k}}_{0}=0.5$, $\bar{I}=800$, $f_{\max}=0.1$, $\bar{\Delta }=0.1$. Similarly, Figure 2c,d show that when the parameters are set to $\bar{N}=0.2$, χ=0.4, $\chi_{\mathrm{SP}}=0.65$, ${\bar{A}}_{0}=1$, ${\bar{k}}_{0}=0.5$, $\bar{I}=800$, $f_{\max}=0.1$, $\bar{\Delta }=0.1$, the system exhibits a self-pulsating state. In this state, the light intensity oscillates over time, and the hydrogel volume also undergoes corresponding oscillations. Through continuous simulations, we found that when the parameters satisfy $\bar{N}=0.6∼1$, χ=0∼0.2, the system enters the stable state, whereas when the parameters fall within the range $\bar{N}=0∼0.5$, χ=0.3∼0.5, the system transitions to the self-pulsating state. Material parameters govern the emergence of two distinct motion states in the light-responsive hydrogel system.

### 3.2. Mechanism of Self-Pulsating

**Figure 3:**
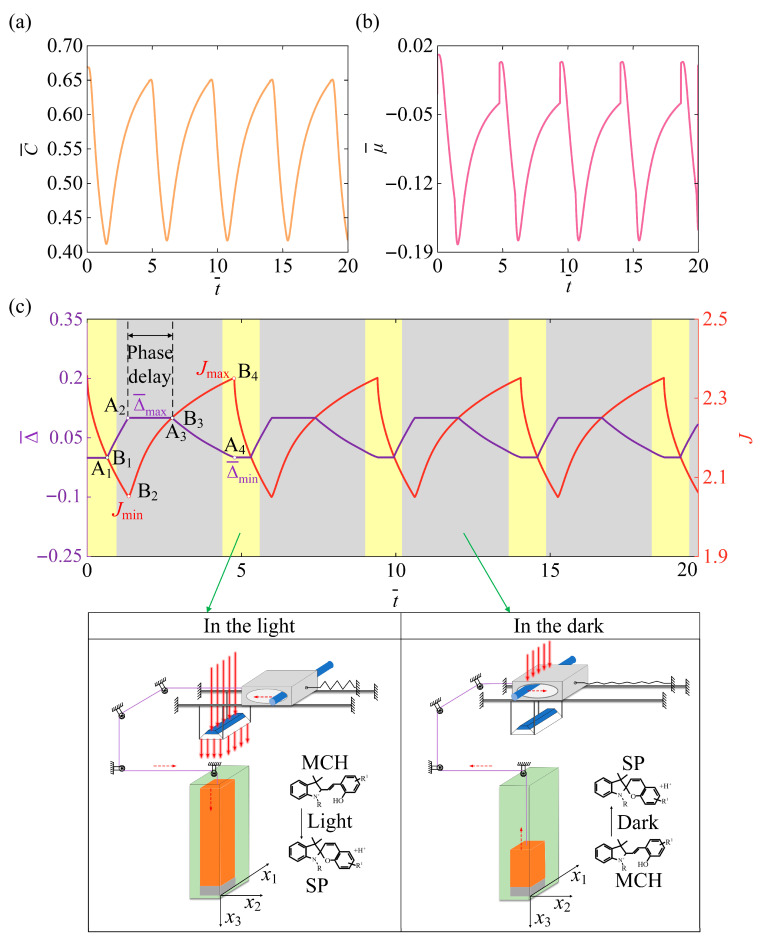
Mechanism of the self-pulsating state in the light-responsive hydrogel system. (**a**,**b**) show the time-course curves of $\bar{C}$ and $\bar{\mu }$ in the self-pulsating state, while (**c**) illustrates the phase diagram between the slider displacement $\bar{\Delta }$ and the hydrogel volume J at different times. In the self-pulsating state, the concentration and chemical potential curves display periodic oscillations, while the slider position and hydrogel volume also oscillate synchronously over time, a clear phase delay is observed between the slider displacement and the hydrogel volume.

Figure 3 shows the mechanism underlying the self-pulsating state of the light-responsive hydrogel system. Figure 3a illustrates the periodic temporal variation in solvent concentration $\bar{C}$ within the hydrogel. These concentration fluctuations regulate the spatial distribution of chemical potential inside the system, thereby driving the hydrogel to undergo cyclic volumetric swelling and deswelling. This mechanism forms the intrinsic physical basis for the emergence of self-excited oscillations. Figure 3b shows that the chemical potential $\bar{\mu }$ exhibits periodic oscillations over time. The chemical potential acts as the direct driving force for the self-excited oscillation: under illumination, the increased chemical potential triggers hydrogel swelling; subsequently, mechanical feedback causes the chemical potential to decrease, inducing deswelling and completing a full cycle of self-excited oscillation. Figure 3c illustrates that both the hydrogel volume J and the slider displacement $\bar{\Delta }$ exhibit oscillatory behavior over the same timescale. During a complete oscillation cycle, illumination induces the conversion of light-sensitive molecules from the MCH form to the SP form, resulting in volume contraction. In contrast, the dark stage corresponds to the shading process, where the molecules revert from the SP form to the MCH form, resulting in volume expansion. The self-pulsating is achieved through volume-feedback-controlled illumination, where the light intensity varies in a square-wave pattern to trigger system responses, causing the hydrogel volume to exhibit a sawtooth-like periodic fluctuation that represents a typical negative-feedback-driven self-pulsating mechanism. A clear phase delay can be observed between the slider displacement and the hydrogel volume. Specifically, the peak of $\bar{\Delta }$ occurs later than that of J, indicating that the mechanical response of the slider lags the hydrogel volume change, thereby revealing the coupled dynamic characteristics of the system.

## 4. Tuning the Self-Pulsating Dynamics

In the discussion above, the motion states of the light-responsive hydrogel system are summarized, which are governed by seven dimensionless parameters. Based on Equations (27)–(33) in Section 2, the parameters $\bar{N}$, χ, $\chi_{\mathrm{SP}}$, ${\bar{A}}_{0}$, ${\bar{k}}_{0}$, $\bar{I}$, $f_{\max}$, and $\bar{\Delta }$ are varied sequentially to analyze their relationships with the self-pulsating state and to investigate their effects on the hydrogel’s self-pulsating behavior. Additionally, the variation in the volumetric oscillation frequency and amplitude under different parameter conditions is studied. The volumetric oscillation frequency f refers to the number of oscillation cycles per unit time, serving as a measure of the system’s oscillation speed. Higher frequency indicates that the hydrogel completes more expansion-contraction cycles in the same time, reflecting a faster system response. Volume oscillation amplitude A refers to the difference between the extreme volume of the hydrogel during each oscillation cycle, reflecting the extent of its expansion and contraction.

### 4.1. Role of the Sliding Displacement

**Figure 4:**
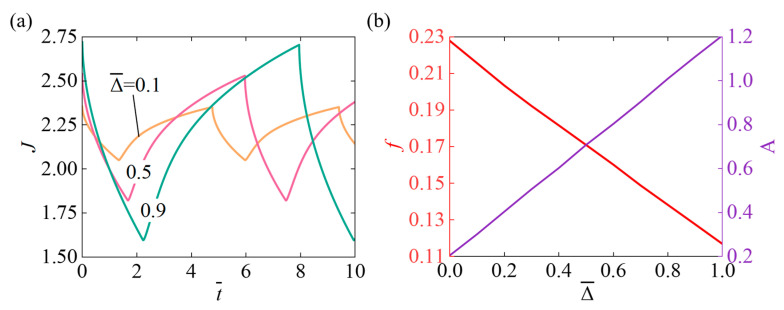
The effect of the sliding displacement $\bar{\Delta }$ under continuous illumination on self-pulsating. (**a**) Volume J (**b**) Frequency f and Amplitude A. Increasing slider displacement $\bar{\Delta }$ results in a simultaneous linear increase in amplitude and a linear reduction in oscillation frequency.

Figure 4 presents the effect of the sliding displacement $\bar{\Delta }$ on the self-pulsating behavior of the light-responsive hydrogel, with parameters set as $\bar{N}=0.1$, χ=0.5, $\chi_{\mathrm{SP}}=0.65$, ${\bar{A}}_{0}=1$, ${\bar{k}}_{0}=5$, $\bar{I}=800$, $f_{\max}=0.1$. Figure 4a shows that as the $\bar{\Delta }$ continues to increase, the system’s oscillation response time gradually becomes longer, while the oscillation amplitude also continuously grows. As the slider displacement increases, the hydrogel requires more time to complete its expansion and contraction, resulting in a longer oscillation response time for the system. Figure 4b shows that the oscillation frequency steadily decreases with increasing $\bar{\Delta }$, and the oscillation amplitude grows progressively as the $\bar{\Delta }$ increases. Because increasing the $\bar{\Delta }$ provides a larger volume freedom that allows the hydrogel to undergo greater expansion and contraction, it enlarges the oscillation amplitude while also requiring more time to complete each oscillation, thereby reducing the system’s oscillation frequency.

### 4.2. Role of Crosslinking Density in the Dried Network Reference State

**Figure 5:**
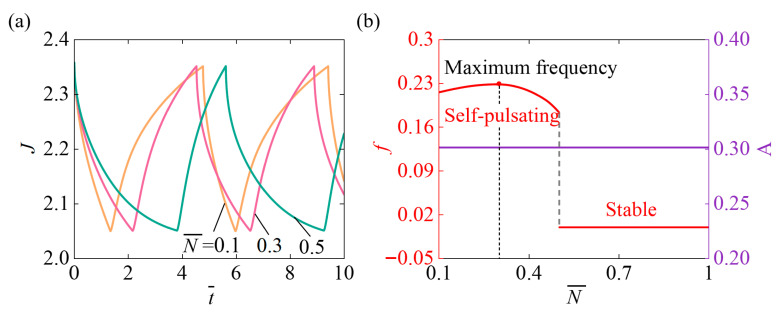
Impact of the crosslinking density in the dry-state network reference state $\bar{N}$ on self-pulsating under continuous illumination. (**a**) Volume J (**b**) Frequency f and Amplitude A. When the $\bar{N}$ gradually augments, the oscillation frequency f first rises steadily, then decreases slowly, and finally drops sharply to zero and stabilizes. Amplitude A remains unchanged as $\bar{N}$ increases.

Figure 5 presents the impact of the crosslinking density in the dry-state network reference state $\bar{N}$ on the self-pulsating under the parameter setting χ=0.5, $\chi_{\mathrm{SP}}=0.65$, ${\bar{A}}_{0}=1$, ${\bar{k}}_{0}=5$, $\bar{I}=800$, $f_{\max}=0.1$, $\bar{\Delta }=0.1$. Figure 5a shows that different values of $\bar{N}$ can change the system’s response time and oscillation period, highlighting the important influence of $\bar{N}$ on the oscillation frequency. Figure 5b demonstrates that as $\bar{N}$ increases, the oscillation frequency first rises gradually, reaches a maximum at $\bar{N}=0.3$, and then declines. When $\bar{N}=0.5$, the frequency abruptly drops to zero, and the system converts from a self-pulsating state to a stable state. Meanwhile, the oscillation amplitude of the system remains unaltered as $\bar{N}$ increases. The non-monotonic change in oscillation frequency originates from the competition between the acceleration of mechanical response and the weakening of chemical driving. At low to moderate crosslinking densities, enhanced network elasticity enables faster volume variations, leading to an increase in oscillation frequency. Whereas at high crosslinking densities, the network becomes overly rigid, the swelling capacity decreases, and the chemical potential driving force is weakened, resulting in a reduced frequency. Increasing $\bar{N}$ only alters the stiffness and equilibrium volume of the hydrogel. However, since the amplitude of the system’s oscillatory behavior is determined by the slider-light interactive mechanism, rather than by the intrinsic swelling capacity of the hydrogel, the oscillation amplitude remains essentially unchanged.

### 4.3. Role of Flory-Huggins Interaction Parameter of Hydrophilic Polymers

**Figure 6:**
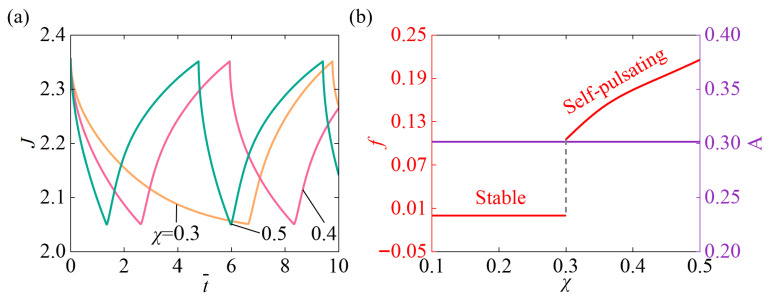
Impact of the Flory–Huggins interaction parameter of the hydrophilic polymer χ on self-pulsating under continuous illumination. (**a**) Volume J (**b**) Frequency f and Amplitude A. With increasing χ, the f first approaches a constant value and then rises steadily, while A shows no change.

Figure 6 presents the impact of the χ on the self-pulsating within the parameter setting $\bar{N}=0.1$, $\chi_{\mathrm{SP}}=0.65$, ${\bar{A}}_{0}=1$, ${\bar{k}}_{0}=5$, $\bar{I}=800$, $f_{\max}=0.1$, $\bar{\Delta }=0.1$. Figure 6a indicates that as χ increases, both the system’s response time and oscillation period gradually shorten. This is because a higher χ reduces the compatibility between the hydrogel and the solvent, resulting in a stronger driving force and more pronounced volume changes. Combined with continuous illumination, the swelling and contraction processes of the system accelerate, thereby shortening the response time and oscillation period. Figure 6b depicts that as χ continues to increase, initially, the system stays in a stable state with near-zero oscillation frequency. When χ=0.3, the system activates the self-pulsating state, and the frequency begins to increase continuously. Meanwhile, the oscillation amplitude of the system remains no change as χ increases. At low χ values, the polymer and solvent are highly compatible, and the hydrogel system is in a thermodynamically stable state, with smooth volume and concentration changes. The system response is weak, making periodic oscillations difficult, resulting in a zero oscillation frequency. Once the parameter exceeds a critical threshold, the nonlinear feedback mechanism triggers self-excited oscillations. The frequency then increases with the strengthening of the driving force. Increasing χ alters the hydrogel’s equilibrium volume. However, since the self-pulsating amplitude is dominated by the slider and light feedback mechanism, the system’s oscillation amplitude remains essentially unchanged.

### 4.4. Role of Flory-Huggins Interaction Parameter of Hydrophilic SP

**Figure 7:**
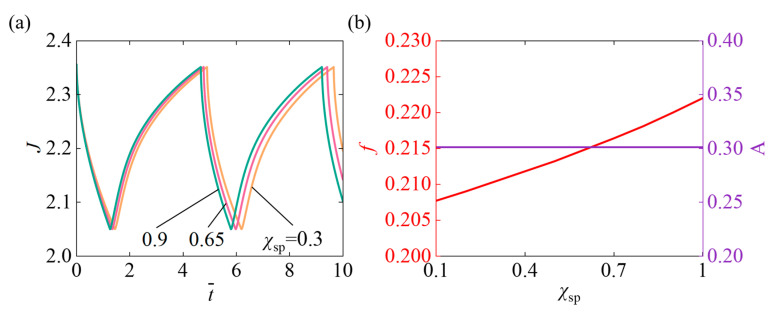
Influence of the Flory-Huggins interaction parameter of hydrophilic SP $\chi_{\mathrm{SP}}$ on self-pulsating under continuous illumination. (**a**) Volume J (**b**) Frequency f and Amplitude A. With increasing $\chi_{\mathrm{SP}}$, the f steadily rises, but A tends toward a horizontal line.

Figure 7 investigates the impact of the Flory-Huggins interaction parameter of hydrophilic SP $\chi_{\mathrm{SP}}$ on the self-pulsating when $\bar{N}=0.1$, χ=0.5, ${\bar{A}}_{0}=1$, ${\bar{k}}_{0}=5$, $\bar{I}=800$, $f_{\max}=0.1$, $\bar{\Delta }=0.1$. Figure 7a illustrates that as $\chi_{\mathrm{SP}}$ increases, the system’s response time and oscillation period continuously shorten. A larger $\chi_{\mathrm{SP}}$ reduces the affinity between the hydrogel and solvent, strengthens the driving force, and accelerates the volume response. Under continuous illumination, the system reacts faster, leading to a shorter oscillation period. Figure 7b reveals that as the $\chi_{\mathrm{SP}}$ increases, the system’s oscillation f continuously rises, and A tends toward a horizontal line. The increase in $\chi_{\mathrm{SP}}$ enhances the nonlinear feedback effect within the system, making the hydrogel’s volume changes more sensitive to light stimulation. This shortens the swelling-contraction cycle and accelerates the oscillation, resulting in a higher oscillation frequency.

### 4.5. Role of Initial Absorption Coefficient

**Figure 8:**
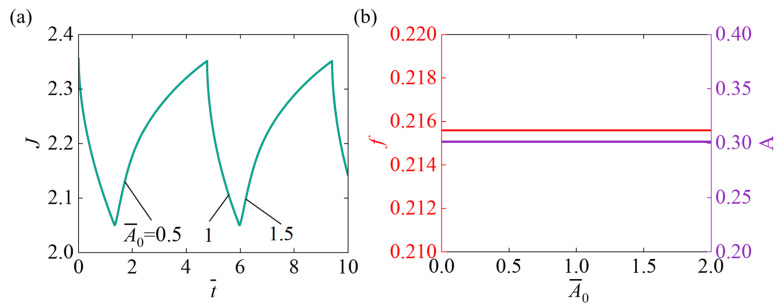
Impact of the initial absorption coefficient ${\bar{A}}_{0}$ on self-pulsating under continuous illumination. (**a**) Volume J (**b**) Frequency f and Amplitude A. As the ${\bar{A}}_{0}$ increases, the oscillation frequency f and amplitude A of the system remains essentially unchanged. With increasing ${\bar{A}}_{0}$, both the system’s oscillation f and A stay nearly constant.

Figure 8 describes the impact of the initial absorption coefficient ${\bar{A}}_{0}$ on the self-pulsating, with parameters set as $\bar{N}=0.1$, χ=0.5, $\chi_{\mathrm{SP}}=0.65$, ${\bar{k}}_{0}=5$, $\bar{I}=800$, $f_{\max}=0.1$, $\bar{\Delta }=0.1$. Figure 8a indicates that as ${\bar{A}}_{0}$ increases, the system’s response time and oscillation period remain unchanged. Although a larger ${\bar{A}}_{0}$ enhances light absorption, the system’s response rate is primarily governed by internal kinetics and diffusion limitations, resulting in stable response time and oscillation period. Figure 8b illustrates that as ${\bar{A}}_{0}$ increases, the system’s oscillation frequency and amplitude approach a horizontal line. Although a higher initial absorption coefficient enhances light energy uptake, the oscillation frequency is primarily limited by internal kinetic processes, such as solvent diffusion rate, chemical reaction rate, and polymer network restructuring, which play a dominant role in determining the oscillation frequency.

### 4.6. Role of Ring-Opened Reaction Rate

**Figure 9:**
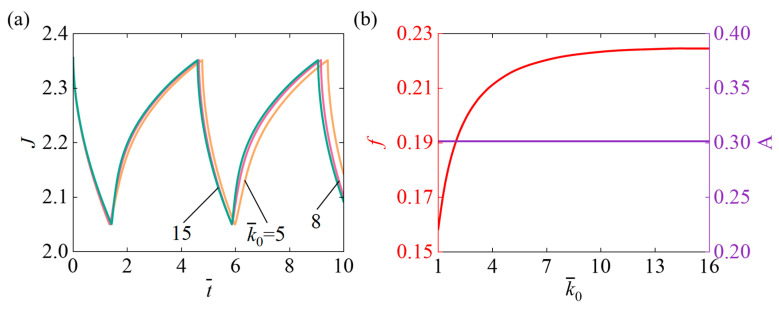
Effect of the ring-opened reaction rate ${\bar{k}}_{0}$ on self-pulsating under continuous illumination. (**a**) Volume J (**b**) Frequency f and Amplitude A. With increasing ${\bar{k}}_{0}$, the f rises rapidly at first and then gradually stabilizes, while the oscillation amplitude remains unchanged.

Figure 9 describes the impact of the ${\bar{k}}_{0}$ on the self-pulsating within the parameter setting $\bar{N}=0.1$, χ=0.5, $\chi_{\mathrm{SP}}=0.65$, ${\bar{A}}_{0}=1$, $\bar{I}=800$, $f_{\max}=0.1$, $\bar{\Delta }=0.1$. Figure 9a highlights that as parameter ${\bar{k}}_{0}$ increases, the system’s response time and oscillation period gradually decrease. The increase in ${\bar{k}}_{0}$ accelerates the conversion of light-responsive molecules and the hydrogel’s volume change, strengthening the feedback mechanism and leading to a significant shortening of both the response time and oscillation period. Figure 9b illustrates that as ${\bar{k}}_{0}$ increases, the system’s oscillation frequency first rises and then stabilizes, and oscillation amplitude remains unaltered. Increasing ${\bar{k}}_{0}$ enhances the system’s nonlinear dynamics, facilitating a rapid transition into the oscillatory state and a swift rise in frequency. However, once the rate exceeds a certain threshold, other kinetic processes become limiting factors, and the oscillation frequency gradually levels off. Increasing the ring-opened reaction rate affects the hydrogel’s response speed but does not alter the oscillation amplitude, because the amplitude is determined by the slider-light feedback mechanism.

### 4.7. Role of Light Intensity

**Figure 10:**
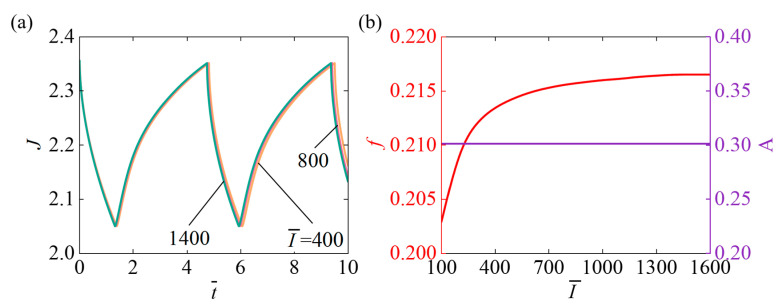
Under continuous illumination, the impact of light intensity $\bar{I}$ on self-pulsating. (**a**) Volume J (**b**) frequency f and Amplitude A. With increasing $\bar{I}$, the f first rises rapidly and then slowly stabilizes. Still, the oscillation A remains constant.

Figure 10 describes the impact of light intensity $\bar{I}$ on the self-pulsating within parameters setting $\bar{N}=0.1$, χ=0.5, $\chi_{\mathrm{SP}}=0.65$, ${\bar{A}}_{0}=1$, ${\bar{k}}_{0}=5$, $f_{\max}=0.1$, $\bar{\Delta }=0.1$. Figure 10a displays that as $\bar{I}$ increases, the system’s response time and oscillation period gradually decrease. This is attributed to the stronger illumination, which enhances the driving force of oscillation and thereby increases the system’s dynamic response speed. Figure 10b presents that as parameter $\bar{I}$ continues to increase, the system’s oscillation frequency initially rises rapidly and then levels off, and oscillation amplitude remains constant. At the early stage of increasing $\bar{I}$, the system gains more energy, the driving force grows, and processes such as swelling and contraction accelerate, leading to a continuous increase in oscillation frequency. However, when the $\bar{I}$ becomes too high, the system’s overall dynamics reach a balanced state, and the oscillation frequency stabilizes without further significant increase.

### 4.8. Role of the Proportion of All Light-Sensitive Molecules Relative to the Total Polymer Amount

**Figure 11:**
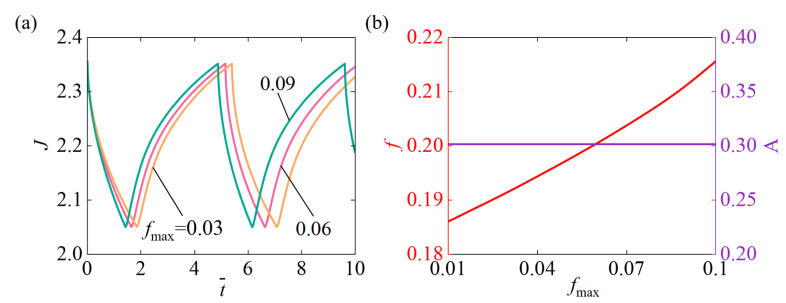
The impact of the proportion of all light-sensitive molecules to the total polymer amount $f_{\max}$ under continuous illumination on self-pulsating. (**a**) Volume J (**b**) Frequency f and Amplitude A. With increasing $f_{\max}$, the oscillation f of the volume also rises. However, the oscillation amplitude is maintained.

Figure 11 investigates the impact of the $f_{\max}$ on the self-pulsating, within parameters setting $\bar{N}=0.1$, χ=0.5, $\chi_{\mathrm{SP}}=0.65$, ${\bar{A}}_{0}=1$, ${\bar{k}}_{0}=5$, $\bar{I}=800$, $\bar{\Delta }=0.1$. Figure 11a illustrates that with the continuous increase of $f_{\max}$, both the oscillation response time and the period of the slider system decrease significantly. Increasing $f_{\max}$ accelerates the system’s response, leading to shorter oscillation times and periods. Figure 11b mainly illustrates that with increasing $f_{\max}$, the system’s oscillation frequency rises, while the amplitude remains constant. As $f_{\max}$ increases, the system absorbs light more efficiently and drives changes in chemical potential more effectively, thereby shortening the characteristic timescale of volume evolution and leading to an increase in oscillation frequency. Increasing $f_{\max}$ enhances the hydrogel’s response speed but does not change the oscillation amplitude.

## 5. Oscillating Lateral Stress and Swelling Force

During the self-pulsating process of the light-responsive hydrogel, the swelling force and lateral stress are key mechanical responses induced by the hydrogel’s periodic volume changes. The swelling force generated at the base of the hydrogel layer within the container originates from the osmotic pressure difference during the swelling-contraction cycles, converting light energy into mechanical deformation and demonstrating the hydrogel’s mechanical output capability in the oscillatory state. Lateral stress reflects the internal network’s force distribution under non-uniform swelling conditions, revealing the mechanical response characteristics induced by light-triggered reactions and solvent transport. The temporal evolution of swelling force and lateral stress not only reflects the chemo-mechanical coupling in the oscillatory state but also provides critical insight into the system’s energy conversion and transfer mechanisms. This understanding is important for enhancing actuation performance and expanding applications in micro-pumps, soft actuators, and soft robotics.

**Figure 12:**
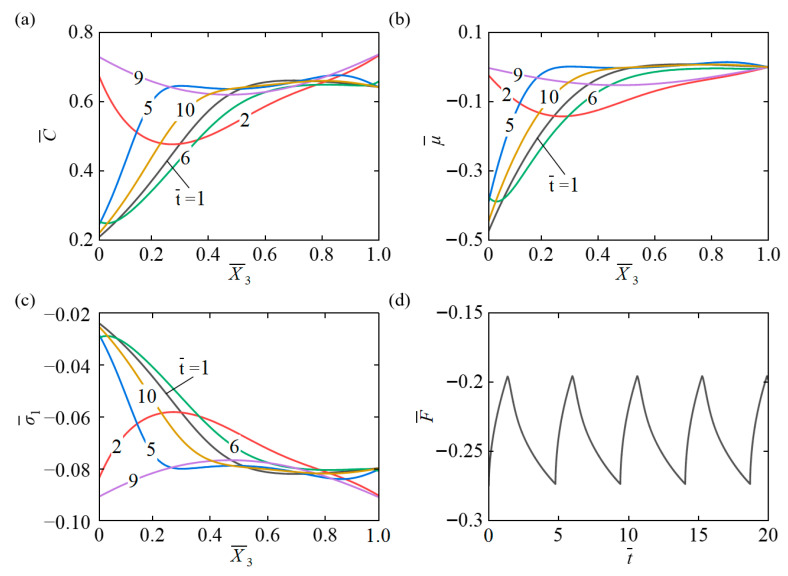
(**a**) Concentration $\bar{C}$, (**b**) chemical potential $\bar{\mu }$ and (**c**) lateral stress ${\bar{\sigma }}_{1}$ temporal-spatial distribution within the hydrogel layer. (**d**) The time evolution curve of the swelling force during the self-pulsating process. The swelling force undergoes periodic fluctuations over time, sharing the same oscillation frequency as the volume.

Using Equations (31) and (33), we determined the time-dependent distribution of lateral stress within the hydrogel layer as well as the corresponding distribution of the swelling force acting on the bottom of the container. Figure 12a illustrates the spatial distribution of solvent concentration $\bar{C}$ along the hydrogel thickness at different time points. It can be clearly observed that at $\bar{t}=2$ and $\bar{t}=9$, the concentration at the upper and lower surfaces of the hydrogel is higher than in the middle region. At other times, the $\bar{C}$ at ${\bar{X}}_{3}=0$ is lower, rapidly increases along the thickness direction, and then gradually levels off. The concentration difference between the interior and surfaces decreases, and the overall profile gradually approaches a uniform distribution. Figure 12b shows the spatiotemporal evolution of the chemical potential $\bar{\mu }$ along the hydrogel thickness. It can be clearly seen that at $\bar{t}=2$ and $\bar{t}=9$, $\bar{\mu }$ is concentrated near the top and bottom surfaces of the hydrogel. At other times, $\bar{\mu }$ is lower at ${\bar{X}}_{3}=0$, initially rises rapidly along the thickness direction, and then gradually levels off. Figure 12c illustrates the temporal evolution of the lateral stress ${\bar{\sigma }}_{1}$ along the hydrogel. It is observed that at $\bar{t}=2$ and $\bar{t}=9$, ${\bar{\sigma }}_{1}$ is concentrated in the middle region of the hydrogel, while at other times, ${\bar{\sigma }}_{1}$ gradually migrates from other regions toward the top surface. Figure 12d expresses that the expansion force $\bar{F}$ exhibits periodic fluctuations during the oscillation process. The dynamic swelling force generated by the self-pulsating hydrogel demonstrates the capability of converting light energy into periodic mechanical output. While this feature suggests potential applicability in microfluidic transport, soft actuation, and related systems, it should be emphasized that the current results are based on numerical simulations. Practical implementation will require careful consideration of system-level factors, including fluid resistance, mechanical losses, and integration constraints.

## 6. Conclusions

To address the long-standing challenge that hydrogels struggle to achieve large-amplitude and stable self-pulsation under constant stimuli, this work proposes a mechanically delayed feedback mechanism based on a slider system, which leverages clearance-induced lag and amplification to convert the subtle deformations of a light-responsive hydrogel into large-amplitude and sustained oscillations. The proposed light-responsive hydrogel system is theoretically demonstrated to exhibit periodic self-pulsating characteristics. Theoretical analysis of the system under continuous illumination reveals that, under appropriate light intensity and material parameters, the hydrogel can rely on its intrinsic feedback and time-delay effects to convert continuous light energy input into regular volume oscillations, thereby achieving swelling-deswelling cycles without external periodic driving. The light-induced contraction process, when coupled with the mechanical motion of the slider and delayed feedback, ultimately triggers and amplifies the self-pulsating behavior.

The dynamic behavior of the light-responsive hydrogel system is jointly influenced by material parameters and light intensity. In the self-pulsating state, the volumetric oscillation frequency increases with the Flory–Huggins interaction parameter of the SP, the Flory–Huggins interaction parameter of the hydrophilic polymer, the ring-opened reaction rate, the light intensity, the proportion of all light-sensitive molecules relative to the total polymer amount, and the sliding displacement. As the ring-opened reaction rate and light intensity continue to increase, the oscillation frequency gradually approaches a steady value. It is noteworthy that the volumetric oscillation frequency does not vary monotonically with the dry-state network reference crosslink density; instead, it exhibits a nonlinear trend of first increasing and then decreasing. As the dry-state network reference crosslink density continues to rise, the system’s oscillation frequency drops suddenly to zero, indicating a shift from self-pulsating to stability. Moreover, the initial absorption coefficient has no significant effect on the oscillation frequency, indicating that changes in this parameter do not influence the response time or oscillation period of the light-responsive hydrogel. In contrast, increasing the slider displacement not only causes the oscillation frequency to decrease linearly but also provides greater volume freedom, allowing the hydrogel to undergo larger expansions and contractions, which results in a linear increase in the oscillation amplitude. Other parameters studied do not affect the oscillation amplitude. The self-pulsating behavior of the hydrogel simultaneously generates stress and swelling force: the internal stress gradually migrates toward the upper surface, while the swelling force exhibits periodic fluctuations synchronized with the volumetric oscillations, demonstrating the characteristic features of self-pulsating.

Future experimental studies are recommended to validate the theoretical predictions presented in this work. From a practical implementation perspective, several key challenges must be addressed. Friction between the slider and substrate may dissipate energy and suppress oscillations, so low-friction or lubricated interfaces are desirable. Fabrication tolerance of the clearance structure is critical, as excessive deviation can weaken the delay-induced feedback. Precise control of hydrogel dimensions and optical alignment is also necessary to avoid light loss and ensure stable performance. Long-term operation may be affected by material fatigue, photobleaching, and solvent evaporation, which could gradually alter system behavior. Overall, this work provides a theoretical framework for understanding delay-induced self-pulsating in light-responsive hydrogels through coupled chemo–mechanical–optical interactions. The findings primarily establish a conceptual design strategy rather than a fully validated experimental system, and are expected to guide future studies toward practical realization.

## Figures and Tables

**Table 1:** Material properties and geometric parameters.

Parameter	Definition	Value	Unit
*N*	crosslinking density in the dry network reference state	0~1.8 × 10^5^	mol/m^3^
*kT*	temperature in the unit of energy	2.7 × 10^2^	J/mol
Ω	volume of one solvent molecule	1.8 × 10^−5^	m^3^/mol
*D*	diffusion coefficient of the solvent	3 × 10^−9^	m^2^/s
*H*	reference thickness of hydrogel layer	0.5	mm
*χ*	Flory–Huggins interaction parameter of hydrophilic polymers	0~0.5	/
$\chi_{\mathrm{SP}}$	Flory–Huggins interaction parameter of hydrophilic SP	0~1	/
$f_{\max}$	proportion of all light-sensitive molecules relative to the total polymer amount	0.01~0.1	/
$A_{0}$	initial absorption coefficient	0~4000	1/m
$k_{c}$	ring-closed rate	0~3.3	1/s
$k_{0}$	ring-opened rate	0~0.3	1/s

**Table 2:** Dimensionless parameters.

Parameter	$\bar{N}$	${\bar{k}}_{0}$	$\bar{I}$	${\bar{A}}_{0}$	χ	$\chi_{\mathrm{SP}}$	$f_{\max}$	$\bar{\Delta }$
Value	0~1	0~20	0~1600	0~2	0~0.5	0~1	0.01~0.1	0~1

## Data Availability

The data supporting the findings of this study and the MATLAB (2024) codes used for the numerical simulations are available from the corresponding author upon reasonable request.
